# Mechanism and Application of Carbon Nanotube Sensors in SF_6_ Decomposed Production Detection: a Review

**DOI:** 10.1186/s11671-017-1945-8

**Published:** 2017-03-09

**Authors:** Xiaoxing Zhang, Hao Cui, Yingang Gui, Ju Tang

**Affiliations:** 1 State Key Laboratory of Power Transmission Equipment and System Security and New Technology, Chongqing University, Chongqing, 400044 China; 2 School of Electrical Engineering, Wuhan University, Wuhan, 430072 China

**Keywords:** CNTs, SF_6_, Decomposition components, Gas response

## Abstract

Carbon nanotubes (CNTs) have aroused extensive attentions as a new category of gas sensor materials owing to their outstanding performance for detecting specific gas among a variety of ones through diverse gas responses. This review summarizes the adsorption mechanism of CNTs and their properties related to the detection of sulfur hexafluoride (SF_6_) decomposed gases that generated in gas insulation switchgear (GIS) of power system. Their performances as sensors of both experimental analysis and theoretical calculation for various kinds of decomposed gases are summarized, and the further research trend on CNTs in the detection of SF_6_ decomposition components is also put forward.

## Introduction

Sulfur hexafluoride (SF_6_) gas is widely applied as electrical insulator as well as arc-quenching medium in gas-insulated switchgear (GIS) of power system due to its excellent chemically inert and remarkable dielectric strength [1, 2]. However, partial discharge (PD) might be occurred around the point where the electric field is intensified in the GIS especially having operated in a long run, contributing to the decomposition of SF_6_. It has been well known that the formation of decomposition products of SF_6_, also named typical components of SF_6_, like SO_2_, H_2_S, SO_2_F_2_, CF_4_, and SOF_2_ [3–5], could be attributed to the discharge-induced SF_6_ decomposition as well as the succeeding reactions with contaminants such as air or water vapor [6, 7]. Previous researches have demonstrated that these typical gases are able to accelerate the rate of equipment corrosion, increasing the possibility of system paralysis [8, 9]. Therefore, the online detection of the gas components in the GIS is essential and significant to estimate the operation state of power system.

Since carbon nanotubes (CNTs) were first discovered in 1991 by Iijima [10], they had been the focus of a series of scientific and engineering areas and even multidisciplinary areas because of their unique physicochemical properties. Specifically, CNT-based gas sensors have received considerable attention resulting from their prominent properties such as faster response, higher sensitivity, and lower operating temperature [11–14]. Single-walled carbon nanotubes (SWCNTs) consist of a single graphite sheet seamlessly wrapped into a cylindrical tube, while multi-walled carbon nanotubes (MWCNTs) comprise an array of such nanotubes [15]. A large number of researches have also been held to detect the SF_6_ decomposition gases using CNT-based sensors, for the purpose of introducing a novel type of sensors that could be employed as an indicator of current state in GIS. To understand the adsorption mechanism of CNTs and exploit new kinds of CNT-based sensors for safe operation of the power system, the effect of CNTs on specific gases is ought to be evaluated both experimentally and theoretically.

Based on these studies, the adsorption and sensing properties of CNTs based materials to SF_6_ decompositions can be understood. However, there still lack of a summary about the whole results in terms of this field, which is significant because it can systemically exhibit the research status, thereby giving an insight on the application of these materials in sensory technology and encouraging continual research in the years to come. That is what we attempt to do in this work. This paper reviews the present state of the application of CNTs including single-walled carbon nanotubes and multi-walled carbon nanotubes in the detection of SF_6_ decomposed components. This review is not to be comprehensive, since our point is on exploiting exceptional properties of CNTs toward the development of newfangled sensing materials in the field of electronic engineering.

## Adsorption Mechanism of CNTs as Sensors

The sensor-related applications of CNTs to detect variant kinds of gas have never failed to be highlighted in recent years. CNTs are supposed to be a new type of adsorbent and hold significant position in carbon-based sensor materials for many reasons. In the first place, they possess chemically inert surfaces and high specific surface area for physical adsorption, directly providing a diversity of well-defined adsorption sites available for adsorbed molecules [16]. Apart from that, different charge distribution resulted from the charge transfer and different adsorption energy attributed to gas morphology coexisted in the adsorption process give the qualitative and quantitative explanation for the increasing or decreasing conductivity in gas adsorption experiment of the CNT sensors [17], thereby differentiating the specific gas from the others.

### Adsorption Sites for Gas on CNTs

**Fig. 1:**
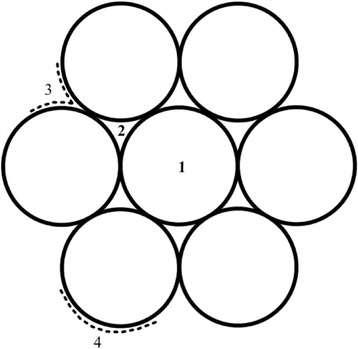
Adsorption sites of CNTs: *1* internal; *2* interstitial channel; *3* external groove; and *4* external surface

It has already been accepted that there are four potential adsorption sites (seen in Fig. 1) in the CNTs [18, 19] for the adsorption of diverse gases: (i) “internal sites”—the hollow interior of every tube; (ii) “interstitial channels”—the hollow channels between individual tubes; (iii) “grooves”—the exterior surface of the tubes, where two adjacent parallel tubes meet; and (iv) “outside surface”—the curved surface of tubes on the outside of the nanotube bundles. That is to say, the gas molecules are able to interact with CNTs through the outer surface of bundles, the interstitial channels between the tubes and the inside of CNTs [20]. According to this fact, a great quantity of studies [21–25] has been carried out in order to confirm the place where the adsorptions of gas molecules with CNTs are most likely to occur.

Though there are several possible adsorption sites, certain gas molecules could only be adsorbed at given ones due to their own properties. Detailedly, CF_4_, one of the decomposed components of SF_6_, could be adsorbed at 133 K on the external sites of closed SWCNTs and on both external and internal sites of open SWCNTs [23, 26], while SF_6_ can only be adsorbed on the outer part of the bundles (grooves and nanotube walls) [22]. Calbi et al. [27] studied adsorption sites and energy barriers near the ends of carbon nanotube bundles to determine their influences on gas adsorption in the interstitial channels between the tubes and obtained two main results. For one, the groove sites exhibit an inverse dependence of capacity on the length of the adsorbed molecule, while the capacity of the internal sites depend inversely on the volume occupied by the molecule, both experimentally and theoretically. For another, although opening the nanotubes could somewhat increase the adsorption rate at the entrance of the channel, the adsorptions on the external grooves are much faster than that of interstitial channels.

Since the external sites are directly exposed to adsorbed gas molecules, the adsorbent process has to be proceeded on the outside sites of the CNTs and then continues to the sites at the interior by diffusion [28, 29]. Therefore, it is unsurprisingly to draw a conclusion that the adsorption at external sites (grooves and outer surfaces) gets much quicker equilibrium in comparison to internal sites (interstitial channels and inside the tubes) under the same pressure and the temperature condition, as the latter needs to take more time to interact.

### Adsorption Energy of Gas on CNTs

Every adsorption site has relevant adsorption energy (*E*_ad_), which is calculated by the following equation, expressed as:

1 $$ {E}_{\mathrm{ad}}={E}_{\mathrm{CNT}/\mathrm{molecule}}-{E}_{\mathrm{CNT}}-{E}_{\mathrm{molecule}} $$

The experimental results of Kondratyuk et al. [24] have presented that the highest energy adsorption site is ascertained as the interior sites of nanotube, followed by groove sites on the outside of the adjoined bundles, and finally the external surface of nanotubes. Based on the work of Williams et al. [30, 31], the *E*_ads_ between CNTs and H_2_ at different sites follow the order: *E*_ad_ (channels) > *E*_ad_ (grooves) > *E*_ad_ (internal) > *E*_ad_ (surfaces). It should be mentioned that the larger the *E*_ads_ is, the harder the adsorption would occur; conversely, the more negative it is, the more spontaneously the process would happen, which basically gets in accordance with the deduction mentioned above.

## Application of CNT Sensors

Given its own unique properties analyzed above, the CNTs are frequently employed as adsorbents to adsorb certain pollutants and even prepared as sensors to implement some industry application, one is to detect the SF_6_ decomposition components for the sake of guaranteeing the safety operation of the power system. In recent years, both intrinsic CNTs that possess unique advantages to be adopted as sensors and modified CNTs that could effectively improve the adsorption amount and selectivity of the targeted gases are investigated to analyze their sensitivity to SF_6_ decomposed products. The dopants are not restricted to functional groups, but are evident among metal and nonmetal atom(s). CNTs can be modified by self-assembly of molecules or macromolecules to CNTs forming thermodynamically stable structures by noncovalent interactions such as hydrogen bond, π-π stacking, electrostatic forces, hydrophobic interactions, and van der Waal forces [32]. Meanwhile, the experimental and theoretical studies both play a significant role in fundamental and practical point of view because it would shed new light on the response mechanisms of CNT-based sensors to better diagnose the insulation of GIS.

### Theoretical Calculation for CNT Sensors

Theoretical calculation is a needed means to simulate the adsorption of target gases on SWCNT through building model in relevant software, and the most commonly used one is Materials Studio. Through that, the models of individual molecules and SWCNT are able to be set up and optimized to get the most stable state. When it comes to modification, one carbon atom would be substituted by another atom and then relaxed to their most stable geometric structures, so that the certain element-modified nanotube can be obtained [33], or the group is linked on the surface of the tube and then optimized to obtain the group-modified nanotube.

**Fig. 2:**
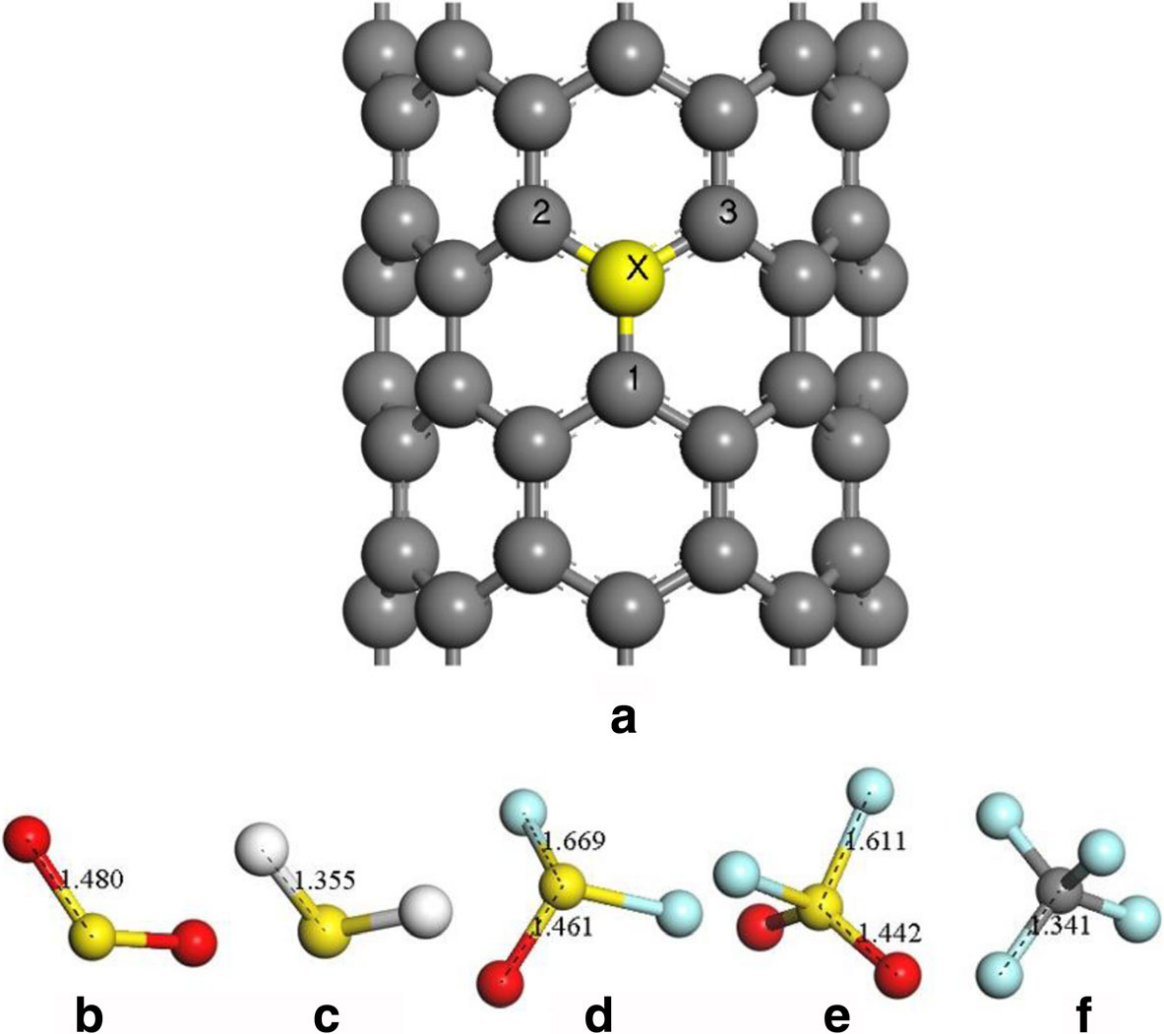
Geometric structure after optimization, **a** instinct SWCNT, **b** SO_2_ molecule, **c** H_2_S molecule, **d** SOF_2_ molecule, **e** SO_2_F_2_ molecule, and **f** CF_4_ molecule. The structural parameters are shown as Å

Figure 2 shows the geometric structures of SWCNT and the main decomposition products of SF_6_ including SO_2_, H_2_S, SOF_2_, SO_2_F_2_, and CF_4_ that have been geometrically optimized, in which the gray, yellow, red, white, and aqua balls represent carbon atom, sulfur atom, oxide atom, hydrogen atom, and fluorine atom, respectively, and the atom smeared by yellow and labeled by X on the sidewall of SWCNT (in Fig. 2a) is the atom to be replaced.

#### Pristine SWCNT

A few studies are performed through Materials Studio to realize the theoretical analysis of adsorption processes between SWCNT and typical gases of SF_6_. Zhang et al. [34] investigated the adsorption characteristic of SWCNT exposed to SF_6_ decompositions and found that the nanotube is more sensitive to SO_2_F_2_ in comparison to H_2_S, SO_2_, or CF_4_ since the electrical conductivity of SWCNT is sharply enhanced after the adsorption of SO_2_F_2_, demonstrating its suitability for preparing sensors to detect SO_2_F_2_. Ding et al. [35] studied the SWCNT gas response to typical SF_6_ decomposition products and concluded that the SWCNT conductivity could be boosted after the adsorption of SOF_2_ or SF_4_, making it possible to exploit new sensors using SWCNTs to diagnose the GIS.

#### Functional Group-Modified SWCNT

Certain oxygen functional groups including −OH and −COOH are intentionally modified during the calculations on the surface of SWCNT toward endowing them better performance on adsorption. Functional groups are capable of transforming the wettability of SWCNT surfaces, enabling them to be more hydrophilic and suitable for adsorbing some low molecular weight and polar compounds [36, 37]. Zhang et al. [38] performed the first principle theory to investigate the gas response of SO_2_F_2_, SOF_2_, SO_2_, and CF_4_ to COOH-SWCNT, with the size of this super lattice 20 Å × 20 Å × 8.5 Å. The generalized gradient approximation (GGA) method was employed to deal with the exchange correlation effects between electrons, and the Perdew-Burke-Ernzerhof (PBE) format was used. They found that the sensitivity of carboxyl-modified SWCNT to these gases keeps to the order: SO_2_ > SOF_2_ > SO_2_F_2_ > CF_4_; thereby, the prepared sensor by doping −COOH onto the CNTs is capable of selectively detecting these typical gases so that to estimate the insulation state of GIS.

**Fig. 3:**
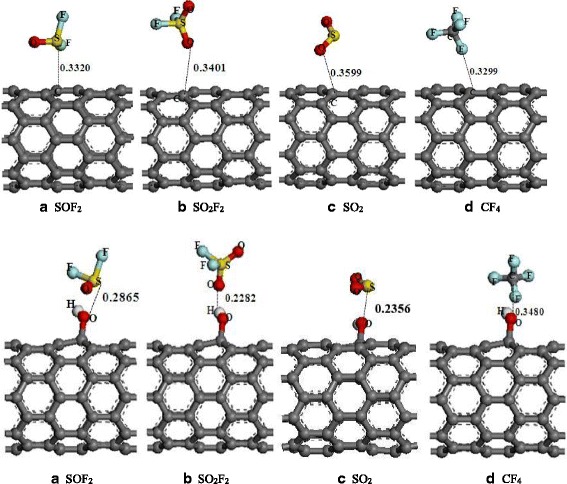
Optimized structures of SWCNT-OH and intrinsic SWCNT interacting with gas molecules. **a** SOF_2_, **b** SO_2_F_2_, **c** SO_2_, and **d** CF_4_

In the theoretical research [39], the intrinsic SWCNT and hydroxyl-modified SWCNT were employed to simulate their adsorption processes with main decomposed products of SF_6_ (SOF_2_, SO_2_F_2_, SO_2_, and CF_4_) generated by PD. GGA method with the PBE format was employed to deal with the exchange correlation effects between electrons. The energy convergence criterion for geometrical optimization was chosen as 10^−4^ eV, while the energy gradient and atomic offset were set at 0.1 eV/Å and 0.005 Å, respectively. The related simulation configurations are shown in Fig. 3; the electron transfer (*Q*_t_), adsorption energies (*E*_ads_) and lengths (*D*) are given in Table 1, while the energies of HOMO and LUMO for OH-SWCNT before and after interaction with these gases are exhibited in Table 2.

**Table 1:** Electron transfer, adsorption energies, and length of adsorbents and gas molecules

Adsorbate	Molecule	*E*_ad_ (eV)	*Q*_t_ (e)	*D* (nm)
SWCNT-OH	SOF_2_	−2.27	−0.026	0.29
	SO_2_F_2_	−2.18	0.011	0.23
	SO_2_	−2.30	0.129	0.24
	CF_4_	−0.05	−0.003	0.35
Intrinsic SWCNT	SO_2_F_2_	−2.15	−0.007	0.34
	SO_2_	−2.23	0.054	0.36
	CF_4_	−0.02	−0.003	0.33

**Table 2:** HOMO and LUMO values of SWCNT-OH before and after adsorption

Adsorptions	HOMO (ha)	LUMO (ha)	*E*_g_ (eV)*
SWCNT-OH	−0.1652	−0.1589	0.1714
SWCNT-OH/SOF_2_	−0.1743	−0.1680	0.1714
SWCNT-OH/SO_2_F_2_	−0.1703	−0.1639	0.1741
SWCNT-OH/SO_2_	−0.1753	−0.1691	0.1687
SWCNT-OH/CF_4_	−0.1649	−0.1585	0.1741

*E_g_ = |HOMO-LUMO| × 27.212 eV

From Table 1, all the adsorption energies present negative, reflecting that these reactions occur spontaneously. Moreover, the more negative *E*_ad_ and shorter *D* for the adsorptions of adsorbate with OH-SWCNT compared with those with intrinsic SWCNT indicate that the sensitivity of OH-SWCNT to these gases is better than that of SWCNT without OH group. Additionally, the energy gap (*E*_g_) applied to determine the difficult degree of the electron transfer and the conductivity change of OH-SWCNT was also taken into consideration. As for the adsorptions of SOF_2_, SO_2_F_2_, and CF_4_, the *E*_g_ keep unchanged or a slight increase, indicating that this material is insensitive to them. Conversely, the *E*_g_ declines sharply after the SO_2_ adsorption, demonstrating that the conductivity change of sensors would be high to this gas, which further proves that the OH-SWCNT-prepared sensors are sensitive to SO_2_. Overall, the results and analyses above indicate that the OH-SWCNT manifests the highest gas response to SO_2_, followed by SOF_2_ that is slightly higher than SO_2_F_2_, while the lowest one comes to CF_4_.

#### Metal/Nonmetal-Doped CNTs

The adsorption ability of CNTs tends be changed through doping metal or nonmetal on their sidewall. The dopant atom(s) coupled with the carbon cage can constitute a mutual area which would exert great influence on adsorption behavior of the as-produced CNTs for gases. As a result, they are usually deemed as proper materials for gas sensors. The adsorption properties of CNTs that doped metal like Pt [40], Au [41], Pd [42], Ni [43], Al [44], and K [45] and nonmetal including B [46] and N [46, 47] have been investigated. In small gas molecule adsorption configurations, the dominate interaction between metal CNT and gas molecules comes to the site for electrode CNT, i.e., the most favorable adsorption site for small gases on metal CNTs is at the electrode [48]. Such provides sensing mechanisms for decompositions adsorbed on metal-doped CNTs. Zhang et al. [49] studied the gas sensitive response of Pd-SWCNT sensors to five kinds of SF_6_ decomposition gases (SO_2_F_2_, SOF_2_, SO_2_, H_2_S, and CF_4_) through density functional theory and found that once exposed to adsorbed gases, the conductivity of nanotube would increase in the following order: SO_2_ > SOF_2_ > H_2_S, decrease when adsorbed by SO_2_F_2_, and retain invariant in terms of CF_4_. According to the theoretical calculations [50] that apply Au-SWCNT to study its responses to H_2_S and SO_2_, the results manifested that the Au-doped SWCNT has a better sensitivity than intrinsic SWCNT that is without any dopant. Simultaneously, the Au-doped one possesses rewarding responses to the two analytes. That is, plentiful electrons transfer to SO_2_ from Au-SWCNTs when putting the SO_2_ molecule near the surface of nanotube, causing a rise in tube conductivity; in contrast to the H_2_S adsorption, the electrons shift from H_2_S to Au-SWCNT, resulting in the declining conductivity of tube. In that case, two types of SF_6_ specific gases, SO_2_ and H_2_S, are able to be detected by Au-SWCNT-based sensors selectively.

In the theoretical analyses [51], the model of B-doped SWCNT was built to investigate its adsorbing interactions to SO_2_F_2_ and derived several conclusions. First of all, the nanotube tends to be a P-type semiconductor and its conductivity raises after B atom has doped on the SWCNT. Besides, chemisorption occurs between B-doped nanotube and SO_2_F_2_, and during which, the conductivity witnesses a remarkable growth, which presents higher sensitivity to SO_2_F_2_ in comparison with SWCNT. Most of all, the B-SWCNT has little response to SF_6_ on the basis of simulation.

**Fig. 4:**
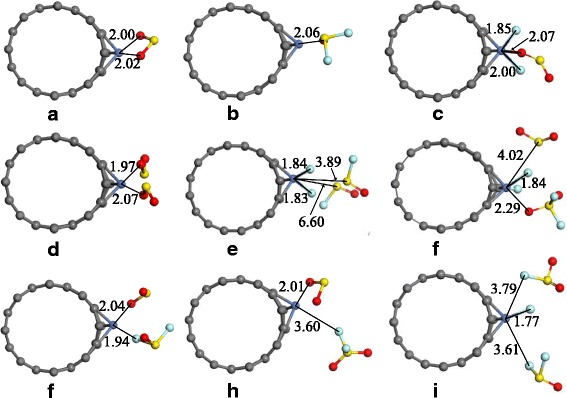
Most stable geometries of gas molecules interacting with Ni-doped SWCNTs (distances in Å)

Zhang et al. [52] employed Ni-doped (8, 0) SWCNTs (64 C atoms and 1 Ni atom) to analyze its sorption to SO_2_, SOF_2_, and SO_2_F_2_ base on first principle theory. The Brillouin zone was performed by the Monkhorst-Pack scheme, sampled into 1 × 1 × 2 k-point [53], and the supercell established for pristine and Ni-doped SWCNTs was restricted to 20 Å × 20 Å × 8.5 Å in the whole calculations. The GGA with the Perdew-Burke-Ernzerhof exchange correlation function in DFT [54, 55], and the basis set employs double numerical plus polarization atomic orbitals. Figure 4 shows that gas molecules approach to the Ni-doped CNTs for adsorption, in which (a), (b) and (c) represent the single molecule adsorbing systems for SO_2_, SOF_2_ and SO_2_F_2_, respectively; (d), (e) and (f) represent the double molecules adsorbing systems for SO_2_, SOF_2_ and SO_2_F_2_, respectively; finally (g), (h) and (i) represent the mixed molecules adsorbing systems for SO_2_&SOF_2_, SO_2_&SO_2_F_2_ and SOF_2_&SO_2_F_2_, respectively. According to these configurations, one can see that through doping the Ni atom on the surface of the nanotube, gas molecules tend to approach the surface of the Ni-doped active site, namely the doped Ni atom provides SWCNT with improved adsorption ability to gas molecules. The banding distance *D*, binding energy *E*_ads_, and charge transfer *Q*_t_ are shown in Table 3, where *D* presents the nearest distance between the gas molecule and the surface of Ni-SWCNT, and *D*_*1*_ and *D*_*2*_ respectively represent the distances of two different molecules. Similarly, the charge transfer from gas molecules to Ni-SWCNTs is labeled as *Q*_t_, and *Q*_t1_ and *Q*_t2_ refer to the charge transfer value of these two different molecules. All the negative values of *E*_ad_ imply that the adsorptions are exothermic and can occur spontaneously.

**Table 3:** Adsorption energy *E*_ads_ and charge transfer *Q*_t_ from adsorbed gas molecules to Ni-SWCNTs

System	*D*_1_ (Å)	*D*_2_ (Å)	*Q*_t1_ (e)	*Q*_t2_ (e)	*E*_ads_ (eV)
SWCNTs/SO_2_	2.00	\	−0.35	\	−1.13
SWCNTs/SOF_2_	2.06	\	−0.06	\	−0.49
SWCNTs/SO_2_F_2_	2.07	\	−0.94	\	−1.93
SWCNTs/2SO_2_	1.97	2.07	−0.24	−0.08	−1.50
SWCNTs/2SOF_2_	3.89	6.60	−0.51	−0.50	−1.79
SWCNTs/2SO_2_F_2_	2.29	4.02	0.06	−0.83	−2.16

\ means there has no results for related D_2_ or Q_t2_

**Fig. 5:**
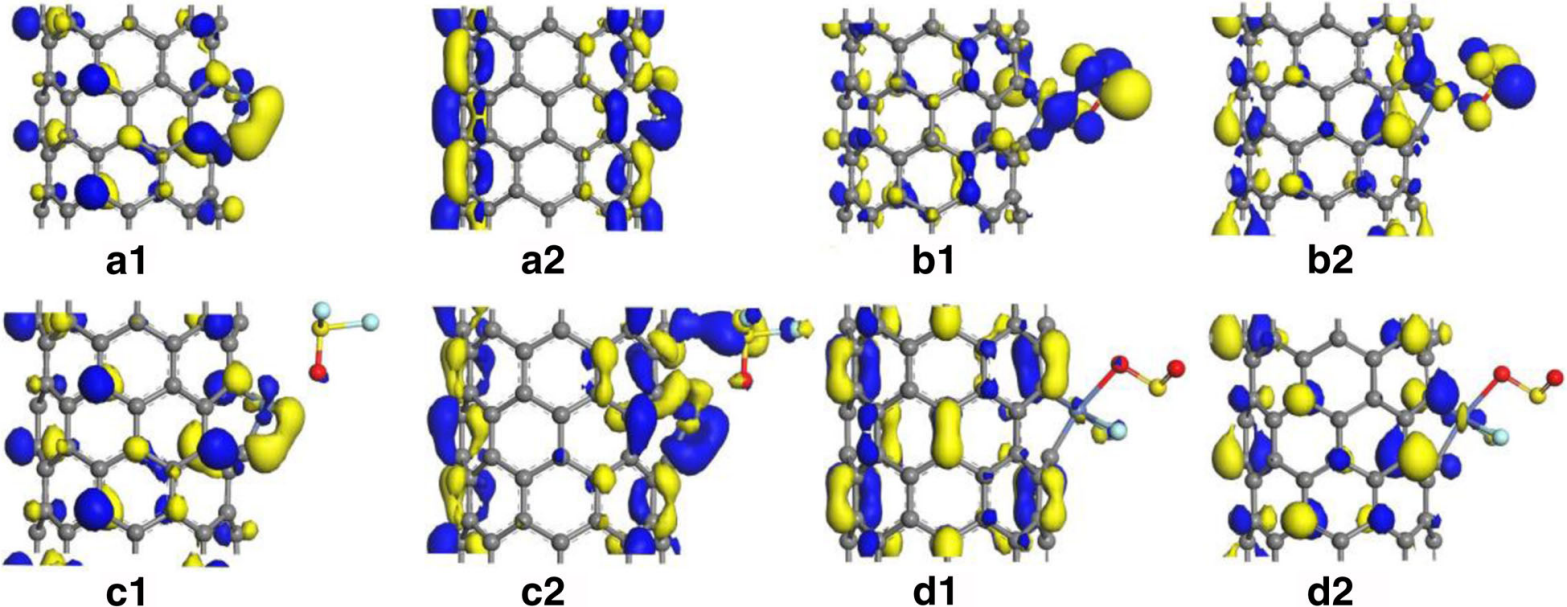
The HOMO and LUMO: **a1** and **a2** intrinsic Ni-SWCNTs, **b1** and **b2** SO_2_-adsorbed Ni-SWCNTs, **c1** and **c2** SOF_2_-adsorbed Ni-SWCNTs, and **d1** and **d2** SO_2_F_2_-adsorbed Ni-SWCNTs

**Fig. 6:**
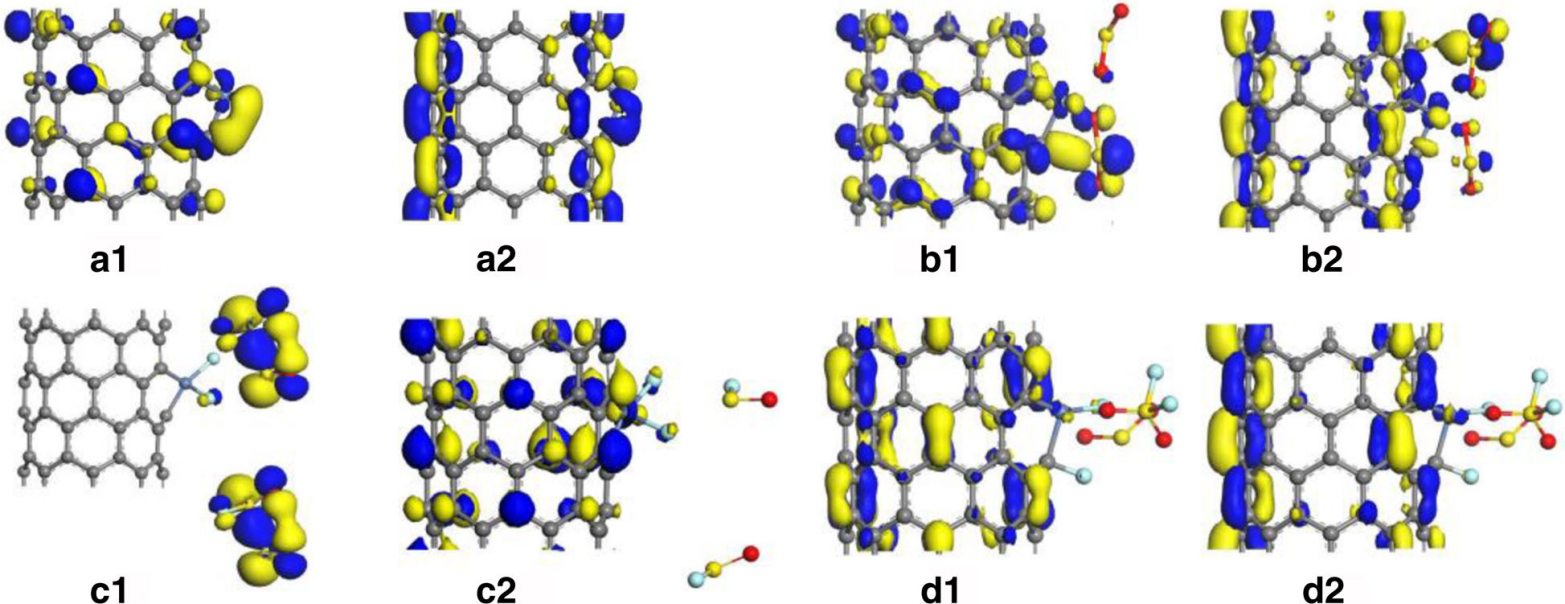
The HOMO and LUMO: **a1** and **a2** intrinsic Ni-SWCNTs, **b1** and **b2** double SO_2_ adsorption, **c1** and **c2** double SOF_2_ adsorption, and **d1** and **d2** double SO_2_F_2_ adsorption

For gases detection, the mechanism of chemical gas sensors discussed above is based on the conductivity change of gas-sensing materials when the gas molecules interact with its surface. Frontier molecular orbital theory is an effective way to explain the change of conductivity upon gas adsorption process. Figures 5 and 6 separately represent the highest occupied molecular orbital (HOMO) and lowest unoccupied molecular orbital (LUMO) of individual gas molecule and double molecules of SO_2_, SOF_2_, and SO_2_F_2_. Due to the effect of Ni doping, the HOMO and LUMO transfer to the SO_2_ adsorption site as shown in Figs. 5b and 6b, just that the LUMO slightly changes to the SOF_2_ in Figs. 5c and 6c and few orbital surrounding the adsorbed SO_2_F_2_ in Figs. 5d and 6d. Thereby, they finally drew the conclusions that the conductivity of nanotube grows up in the following order: SO_2_ > SOF_2_, after their adsorption, while slightly drops after adsorbing SO_2_F_2_ as a result of its chemisorption on CNTs.

**Fig. 7:**
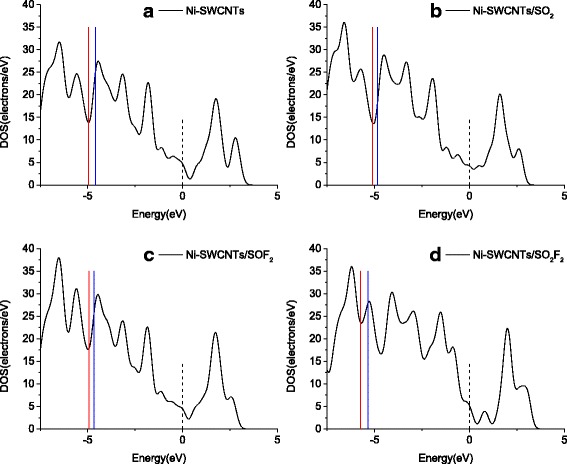
DOS for different adsorbing systems. *Blue line* represents HOMO, *red line* represents LUMO, and *dash line* represents Fermi Level. **a** Ni-SWCTs, **b** Ni-SWCNTs/SO_2_, **c** Ni-SWCNTs/SOF_2_, and **d** Ni-SWCNTs/SO_2_F_2_

To understand the electronic behavior during the adsorbing process, density of state (DOS) that can directly observe the conductivity change is taken into consideration (seen in Fig. 7). In terms of Ni-doped SWCNTs, Bak et al. [56] reported that mesoporous nickel/carbon nanotube hybrid material presented high conductivity, which is in agreement with our calculation that DOS for Ni-SWCNT shown in Fig. 7a has good continuity. Comparing DOS for SO_2_/Ni-SWCNT system (in Fig. 7b) and insolated Ni-SWCNTs, one can find that significant change occurs near the Fermi energy, leading to the increasing conductivity of the system after adsorbing SO_2_. In the case of SOF_2_ adsorption configuration as depicted in Fig. 7c, although the DOS increases slightly near the Fermi level, it fails to contribute to the remarkable change for conductivity. In Fig. 7d where the DOS for SO_2_F_2_ system is shown, it can be observed that the DOS has an increase below the Fermi level and has a decrease above the Fermi level, which is attributed to the chemisorption of Ni-doped complex for this gas. Given these analyses, it would be explicit to comprehend the confirmed conclusions above.

Table 4 shows the overall adsorption simulation results of SWCNT to SF_6_ decomposed gases. It could be easily found that the SWCNT without any treatment is more likely to response to SO_2_F_2_, while modified SWCNT, in spite of functional group doped or metal/nonmetal doped, tends to adsorb SO_2_. Besides, two things should be mentioned that (i) modified CNT have better sensitivity than the non-treated one and (ii) the simulate results present that SWCNT has little response to SF_6_.

**Table 4:** Overall adsorption simulation results of adsorbed gases on CNT

Kinds of CNT	Adsorbed gases					Optimal sensitivity
	SO_2_	H_2_S	SO_2_F_2_	SOF_2_	CF_4_	
Intrinsic SWCNT	√	√	√	√	√	SO_2_F_2_
SWCNT-COOH	√		√	√	√	SO_2_
SWCNT-OH	√		√	√	√	SO_2_
SWCNT-Pd	√	√	√	√	√	SO_2_
SWCNT-Au	√	√				SO_2_
SWCNT-Ni	√		√	√		SO_2_
SWCNT-B			√			–

### Experimental Analysis for CNT Sensors

Based on experiment evidences, it would be more visual and simpler to comprehend the adsorption process between CNTs and adsorbed gases. In this section, we prefer to introduce the concerned methods and results studied previously.

#### Preparation Methods and Measurement of CNT Sensors

**Fig. 8:**
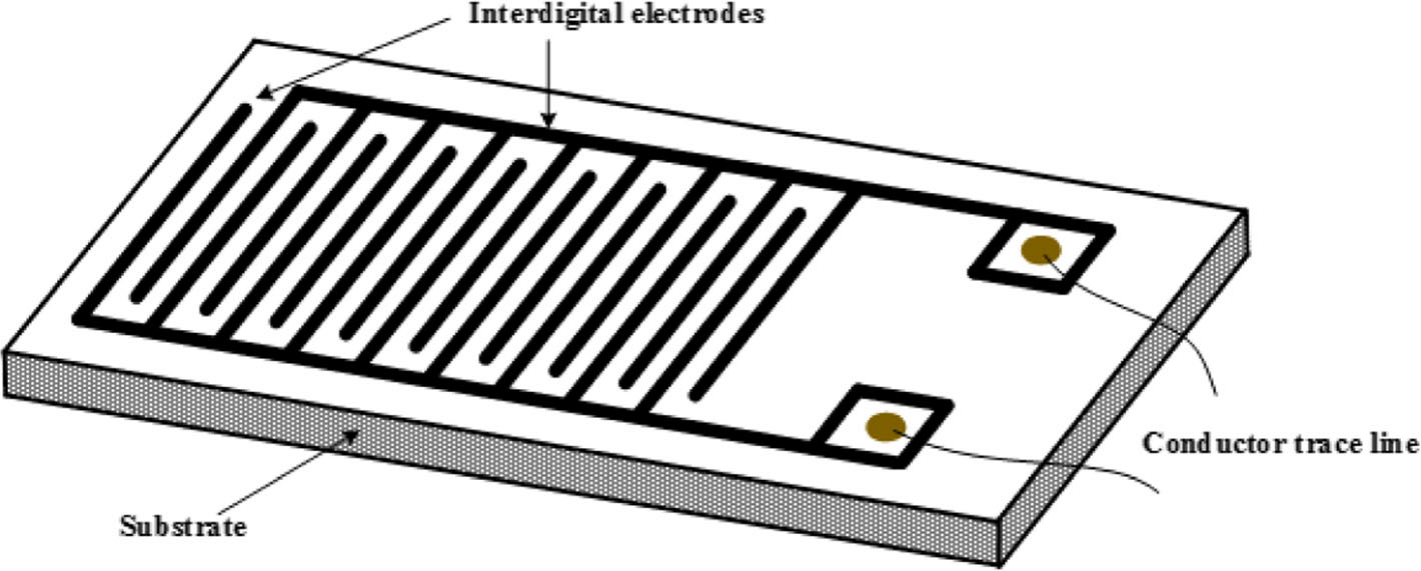
Geometrical morphology of film sensor

Li et al. [57] introduced a facile method for preparing MWCNT sensors. The MWCNTs and palladium chloride were mixed by magnetic stirring, and the NaBH_4_ solution was added dropwise under ultrasonic treatment for the reduction of Pd^2+^, to gain the aqueous dispersion of the nanocomposite of MWCNTs and Pd. The nanocomposite was then ultrasonicated for 2 h and deposited onto an electrode by dip coating with an automatic dip-coating machine, and then dried in air to obtain a gas sensor. Zhang et al. [58] designed an interdigital film sensor that etches the copper electrodes with about 30-μm-thick foil and 0.2-mm electrode gap on a substrate as shown in Fig. 8. The so prepared alcohol dispersion of nanotubes is dropped on the interdigital region of the film with a pipette. The nanotube would attach on the film after alcohol volatilized. This step should be repeated for several times until the sensors are finally obtained with a uniform, dense, and smooth deposition on the surface of films.

The sensitivity is introduced to measure the gas response of sensors, expressed as [59]:

2 $$ S=\frac{\left|{R}_{\mathrm{gas}}-{R}_{\mathrm{sensor}}\right|}{R_{\mathrm{sensor}}} $$

where *R*_gas_ is the steady-state resistance of the sensor in the presence of a given gas concentration and *R*_sensor_ is the baseline resistance of the sensor in dry air. The electrochemical workstation machine is used for continuous monitoring the resistance of the sensors during the measurement process, and the acquired data would be stored in the PC.

#### Pristine CNTs

Jung et al. [60] fabricated SWCNTs gas sensors by alternating current dielectrophoresis, to detect dissociated and oxidized SF_6_ gas species generated by PD in a sealed chamber, leading to two beneficial recoveries. First, SWCNT sensors do not interact with pure SF_6_ but sensitively response to its decomposed and oxidized products. Apart from that, these sensors are renewable through the clearance of fresh air because the physisorption of objects is reversible. As a consequence, the SWNT sensor is a hopeful device for the monitoring of the PD activity inside GIS. Ma et al. [61] investigated the response characteristic of SF_6_ typical gases, i.e., HF, SO_2_F_2_, SOF_2_/S_2_OF_10_, and SO_2_, to SWCNTs and derived that the gas sensor display the most sensitive to SO_2_F_2_, followed by SOF_2_; conversely, SO_2_ and HF impose little effect on the conductivity of the sensors, indicating that this sensor is unlikely to the detection of SO_2_ or HF. They also found that the conductivities of the gas sensors increasingly go up within the duration of PD and almost remain linearly with the accumulation of partial discharge energy until getting saturation.

#### CNTs Modified by Functional Groups

As far as functional groups are concerned, they are able to be formed on the surface of CNTs through oxidation using multiple acids [62, 63], ozone [64, 65], or plasma [66], contributing to, to a large extent, the improvement of the maximum adsorption capability. Lu et al. [67] applied MWCNTs that were oxidized by a series of acids concerning mixed HNO_3_/H_2_SO_4_ (3N + 1S), HNO_3_, KMnO_4_, and NaClO to investigate their effects on the surface characteristic of CNTs. The intrinsic SWCNTs possess the surface area of 435 m^2^/g and 8.35 nm for average pore size, and after oxidation, the surface area and average pore size both occur dramatic drop. Most diameters of intrinsic MWCNTs are in the size >10 nm while of oxidized MWCNTs are in the range of 2–10 nm. It can be concluded that these means made MWCNTs possess a more hydrophilic surface along with a more negatively charged surface, presenting that the physicochemical properties of MWCNTs are dramatically enhanced after oxidation; the MWCNTs (NaClO) appear to be the most effective sorbents, followed by the MWCNTs (KMnO_4_), MWCNTs (HNO_3_), MWCNTs (3N + 1S), and finally the MWCNTs. Zhang et al. [68] employed MWCNTs that are marinated in the solution-mixed HNO_3_/H_2_SO_4_ (1N + 3S) and scattered in the ultrasonic vibration generator for 60 min to fabricate sensors for detecting the SF_6_ decomposition products, and found that not only a large number functional groups, including carboxyl, hydroxyl, and carbonyl, were produced but also many defects are generated at ports and their inter-outer surfaces. It is exactly the growing number of active functional groups and defect positions that apparently increases the gas sensitivity of MWCNT-based sensors.

Another typical study comes to research [69], in which the SWCNTs mixed with potassium hydroxide and proper amount of ethanol are milled for 15 h in a ball milling tank to obtain hydroxyl modification SWCNTs, and SWCNTs are firstly treated with mixture of H_2_O_2_ and H_2_SO_4_ (volume ratio 1:3) and then HNO_3_ and H_2_SO_4_ (volume ratio 1:3) to obtain carboxyl modification SWCNTs, to study the sensitivity and selectivity of SO_2_ and H_2_S. The SWCNT has a purity >90%, length in the range of 1–3 μm, and diameter between 1 and 2 nm, with the surface area of 380 m^2^/g. As far as the response characteristic is concerned, related results indicated that (i) the sensitivity of COOH-SWCNTs to H_2_S and SO_2_ are higher than that of OH-SWCNTs and (ii) the selectivity of both modified SWCNTs to SO_2_ is higher than H_2_S.

According to the Ref [39], the response of OH-SWCNT-based sensors to decomposition gases (500 ppm SO_2_F_2_, SOF_2_, SO_2_, CF_4_) at room temperature and atmospheric pressure were investigated. To prepare OH-SWCNTs, the SWCNTs were added into a beaker which contains ethanol solution, then ultrasonically treated for 1 h. The response curves are calculated based on the above Eq. (1). Results indicated that this type of sensor has the best sensitivity to SO_2_, followed by SOF_2_, and the last one is CF_4_, seeming that such order is related to the number of F atom. For comparison, two concentrations of gases are employed in this work, namely 500 and 250 ppm, and similar response trends can be obtained although the maximum sensitivities are reduced. Overall, it could deduce that OH-SWCNTs have relatively favorable sensitivity to SO_2_, and good selectivity to other gases, which is consistent with the theoretical calculation, bringing about the conclusion that the functional group-modified CNTs are promising materials for sensors to detect SO_2_ and H_2_S.

#### CNTs Modified by Metal/Nonmetal Atom(s)

In the work by Zhang et al. [70], NiCl_2_-doped MWCNT sensors was prepared by ultrasonic NiCl_2_·6H_2_O crystal suspension liquid of carbon nanotubes that pretreated with concentrated acid to test the gas response of SF_6_ decomposition products, and it was derived that the sensors have high sensitivity and fast response to SO_2_F_2_ and SOF_2_, compared to SO_2_.

All the sensitivity and selectivity of so prepared sensors are showed in Table 5. It can be found that SWCNTs could be prepared as sensors to detect SO_2_F_2_, the MWCNTs modified by functional groups are sensitive to H_2_S, and MWCNTs modified by metal have a strong response to SO_2_F_2_. In this way, the selectivity of CNT-based materials in detecting such gases can be preliminarily confirmed.

**Table 5:** Overall experimental results of adsorbed gases on CNTs

Kinds of CNT	Adsorbed gases					Optimal sensitivity
	SO_2_	H_2_S	SO_2_F_2_	SOF_2_	CF_4_	
Intrinsic SWCNTs	√		√		√	SO_2_F_2_
MWCNTs-COOH	√	√				H_2_S
MWCNTs-OH	√	√				SO_2_
MWCNTs-NiCl_2_	√		√	√		SO_2_F_2_

To more clearly know what progresses have been made in this field, we summarized all the results including theoretical studies in parallel with experimental studies of related CNT materials in detecting SF_6_ decompositions, as shown in Table 6. It can be seen that the theoretical and experimental results have good consensus. Specifically, intrinsic CNTs have good sensitivity to SO_2_F_2_, while metal-doped CNTs have good sensitivity to SO_2_. However, response of functional group-modified CNTs to typical gases depends on its geometric structure. Given the various sensitivities of different dopant for CNTs, the selective detection for such gases can therefore be realized.

**Table 6:** Whole applications of CNTs in detecting SF_6_ decomposed gases

	Kinds of CNT	Adsorbed gases					Optimal sensitivity
		SO_2_	H_2_S	SO_2_F_2_	SOF_2_	CF_4_	
Theoretical	Intrinsic SWCNT	√	√	√	√	√	SO_2_F_2_
	SWCNT-COOH	√		√	√	√	SO_2_
	SWCNT-OH	√		√	√	√	SO_2_
	SWCNT-Pd	√	√	√	√	√	SO_2_
	SWCNT-Au	√	√				SO_2_
	SWCNT-Ni	√		√	√		SO_2_
	SWCNT-B			√			SO_2_F_2_
Experimental	Intrinsic SWCNTs	√		√		√	SO_2_F_2_
	MWCNTs-COOH	√	√				H_2_S
	MWCNTs-OH	√	√				SO_2_
	MWCNTs-NiCl_2_	√		√	√		SO_2_F_2_

## Conclusions

The review exhibits the adsorption mechanism and application of CNT-based sensors for detecting the SF_6_ decomposition gases. CNTs possess a large specific surface and a strong van der Waals binding energy, and hence provide well-defined adsorption sites for gas molecules such as interior sites, groove sites, exterior sites, and interstitial sites, enabling the application of CNTs to be an adsorbent to remove some undesirable gases and a sensor to react with target gases reflected by self-changes of physiochemical properties. Most importantly, it has been proved both theoretically and experimentally that the CNTs present low and even little sensitivity to SF_6_, but relatively high sensitivity and selectivity to some of its decomposed components, making it possible to detect the insulation state of the GIS. Much breakthrough has been made during the past decades in application of CNT-based sensors on detecting the GIS operation state. Many CNT sensors have been prepared and found having relatively high sensitivity and selectivity to some SF_6_ decomposed products. Both intrinsic CNTs and modified CNTs including the modification of functional group and metal/nonmetal-doped CNTs have been employed as a theoretical model, helping to understand the adsorption process of CNTs and typical components of SF_6_. However, some types of modified CNTs fail to be adopted as a raw material for the fabrication of sensors to verify the results of simulations. Although its high cost, using CNTs as novel kind of sensors with high sensitivity and quick responses to target gases would offset this demerit, which far into the future can be regarded as desirable material as gas sensors and still be the focus of electric engineers and researchers. Consequently, more effects aimed at the exploitation of new CNT-based gas sensors applied in GIS are ought to be spared. Moreover, given that SF_6_ would be resolved into several kinds of gases under PD and different kind of sensors own different sensitivity to these gases, the sensors’ array should be established in the GIS to realize the highly precise detection of related gases, thus accurately deduce the related insulation faults.
